# Development of loop-mediated isothermal amplification-lateral flow dipstick as a rapid screening test for detecting *Listeria monocytogenes* in frozen food products using a specific region on the ferrous iron transport protein B gene

**DOI:** 10.14202/vetworld.2022.590-601

**Published:** 2022-03-12

**Authors:** Wimvipa Srisawat, Chalermkiat Saengthongpinit, Wirawan Nuchchanart

**Affiliations:** 1 Department of Animal Science, Faculty of Agriculture at Kamphaeng Saen, Kasetsart University, Nakhon Pathom 73140, Thailand; 2 Department of Veterinary Public Health, Faculty of Veterinary Medicine, Kasetsart University, Nakhon Pathom 73140, Thailand; 3 Center for Agricultural Biotechnology, Kasetsart University, Kamphaeng Saen Campus, Nakhon Pathom 73140, Thailand; 4 Center of Excellence on Agricultural Biotechnology, Bangkok 10900, Thailand

**Keywords:** ferrous iron transport protein B gene, frozen food product, *Listeria monocytogenes*, loop-mediated isothermal amplification, loop-mediated isothermal amplification-lateral flow dipstick

## Abstract

**Background and Aim:**

*Listeria monocytogenes* is a critical foodborne pathogen that infects pregnant females and their newborns and older adults and individuals with comorbidities. It contaminates fresh vegetables, fruits, ready-to-eat foods, and frozen food products consumed by individuals. The culture conventional detection methods for *L. monocytogenes* are time-consuming, taking 4 days. This study aimed to describe the development and comparison of loop-mediated isothermal amplification (LAMP)- lateral flow dipstick (LFD), LAMP assay to PCR, and conventional culture for detecting *L. monocytogenes* in frozen food products.

**Materials and Methods:**

Five LAMP primer sets, including F3, B3, forward inner primer, and backward inner primer, were designed from a specific region on ferrous iron transport protein B gene (*feoB* gene) to amplify LAMP products. The DNA probe was created, and the detection limit was determined in pure culture and purified DNA, as well as the detection in 20 frozen food product samples.

**Results:**

The *LMfeoB4* LAMP primer sets and DNA probe were LAMP products amplified at 60°C for 50 min. The specificity of the assay revealed no cross-reactivity with other pathogenic bacteria. The limit of detection (LOD) of the LAMP-LFD and LAMP assays using purified genomic DNA was 219 fg/μL both in LAMP and LAMP-LFD assays. The LOD of LAMP and LAMP-LFD assays in pure culture was 4.3×10^2^ colony-forming unit (CFU)/mL and 43 CFU/mL, respectively. The LOD of the LAMP-LFD assay using artificially inoculated chicken in frozen food samples with pre-enrichment was 3.2×10^2^ CFU/mL. The LAMP-LFD was also more sensitive than the LAMP assay and polymerase chain reaction. Finally, LAMP-LFD revealed no false positives in any of the 20 frozen food product samples.

**Conclusion:**

LAMP-LFD assay using a specific region on the *feoB* gene to detect *L. monocytogenes* was highly specific, sensitive, faster, and convenient, making it a valuable tool for the monitoring and rapid screening of *L. monocytogenes* in frozen food products. This technique is applicable to the development of detection technologies for other pathogens in food products.

## Introduction

The agricultural food industry is crucial to the economies of several countries, including Thailand. The current “food safety” and “traceability” or “from farm to table” issue has piqued the interest of customers both inside and outside the country. *Listeria monocytogenes* can cause agricultural food production problems due to the bacteria contaminating an environment for a long period and growing at low temperature (2-4°C). *L*. monocytogenes can induce listeriosis, which causes meningitis, septicemia, and abortion [1]. According to the European Union One Health 2019 Zoonoses Report, 2621 confirmed cases of invasive listeriosis or infection with *L. monocytogenes*in humans were reported in 2019 [2]. The EU notification rate was 0.46 cases per 100,000 population in 2019, accounting for 17.6% of all fatalities, making *L. monocytogenes* one of the most virulent foodborne pathogens [2]. According to the US Foodborne Diseases Active Surveillance Network (FoodNet), the annual incidence rate per 100,000 cases for *L. monocytogenes* is 0.28 in the general population, 3.73 in pregnant women, and 1.33 in adults over 70 years old; rates are expected to rise from 0.25 to 0.32 in 2030 due to population growth [3]. The FoodNet report in 2019 indicated that there were 25,866 cases of illness, 6164 hospitalizations, and 122 fatalities. The number of laboratory-diagnosed *Listeria* included 134 cases of illnesses, 131 cases of hospitalizations, and 21 cases of death, with an incidence rate of 0.3 cases per 100,000 in the population [4]. The Foodborne Disease Outbreak Surveillance System, United States, 2015 of *L. monocytogenes* reported outbreaks at 35%, with 380 illnesses, 334 hospitalizations, and 74 deaths [5].

There are several methods for conducting an initial inspection and monitoring depth to detect microorganisms in animal products and food ingredients [6]. Typically, manufacturers perform a random check on the bacteria produced by animal feed through microbiological culture media, which requires large volumes and multiple steps. It takes a minimum of 24 h in a culture, making it difficult and time-consuming. The gene amplification technique has a wide range of applications in the laboratory, such as food, molecular research, and detection of contaminants in the environment and food. The most popular gene amplification technique is polymerase chain reaction (PCR) [7,8], which has been used in various patterns, including reverse transcription PCR [9], nested PCR [10,11], multiplex PCR [12], and real-time PCR [13]. Extended genes are employed in the food industry to detect disease-causing contaminants in food. PCR technique is used as the basis to check for about 2-3 h, requiring specific tools with high precision, including PCR and real-time PCR machines, which are expensive. Certain technological procedures use specific tools to detect genes that are increasing. It is impossible to apply this technology in a small lab or the field. In 2000, a report developed with gene expansion technique loop-mediated isothermal amplification (LAMP) by Japanese researchers under the name Tsugunor I Notomi helped to solve critical problems of the PCR technique, the high amplification efficiency under isothermal conditions without the thermal cycler used in PCR. This assay can use the temperature range of 60-65°C to determine the genes that increase the number in the same procedure [14]. Therefore, gene expansion using the LAMP technique is advanced and fast in detecting microorganisms in animal products and feed ingredients [15-17]. The LAMP assay is not required if a thermocycler is used to amplify the genes that improve DNA yield in the same procedure. This technique is suitable for developing countries, small laboratories, and field operations because it is easy and quick [18,19]. Furthermore, to avoid the visualization of LAMP products by agarose gel electrophoresis (AGE), fluorescent DNA dye and chromatographic lateral flow dipstick (LFD) [20,21] format have been applied to reveal LAMP products in a simpler and faster way [22,23].

This study aimed to describe the development and comparison of LAMP-LFD, LAMP assay to PCR, and conventional culture for detecting *L. monocytogenes* in frozen food products.

## Materials and Methods

### Ethical approval

The present study did not involve any invasive procedure, so ethical approval is not required.

### Study period and location

The study was conducted from August 2017 to February 2020 at the Center for Agricultural Biotechnology, Kasetsart University, Kamphaeng Saen Campus, Nakhon Pathom.

### Bacterial strains

*L. monocytogenes* and 21 bacterial strains of non-*L. monocytogenes* were acquired from the Department of Veterinary Public Health, Faculty of Veterinary Medicine, Kasetsart University, Kamphaeng Sean Campus, Thailand (VPHVETKU), Department of Medical Science, Ministry of Public Health, Thailand (DMST) and Microbiology Department, Faculty of Liberal Arts and Science, Kasetsart University, Kamphaeng Saen Campus, Thailand (MICROFLASKU) are presented in Table-1. All strains were transferred from stock to culture in 10 mL of tryptic soy broth (TSB, Difco; USA) and incubated at 37°C for 24 h.

**Table-1:** Bacterial strains used for assays.

Species	Source*
*Listeria monocytogenes*	VPHVETKU
Other *Listeria species*	
*Listeria innocua*	DMST
*Listeria ivanovii*	DMST
*Listeria welshimeri*	DMST
Non-*Listeria* bacterial strains	
*Salmonella* Typhimurium	VPHVETKU
*Salmonella* Enteritidis	VPHVETKU
*Salmonella* Choleraesuis	VPHVETKU
*Salmonella* Typhi 1417	VPHVETKU
*Escherichia coli* ATCC3521	VPHVETKU
*Escherichia coli* 527	VPHVETKU
*Bacillus cereus*	MICROFLASKU
*Bacillus cereus* lab KPS	MICROFLASKU
*Bacillus cereus* 2372	MICROFLASKU
*Staphylococcus aureus* ATCC25923	MICROFLASKU
*Staphylococcus aureus* 2329	MICROFLASKU
*Micrococcus luteus*	MICROFLASKU
*Microbacterium* 1413	MICROFLASKU
*Corynebacterium glutamicum* 461	MICROFLASKU
*Pichia* membranaefaciens 5108	MICROFLASKU
*Rhodotorula mucilaginosa* 5861	MICROFLASKU
*Serratia marcescens*	MICROFLASKU
*Proteus mirabilis*	MICROFLASKU

* VPHVETKU= Veterinary Public Health, Veterinary Medicine, Kasetsart University, DMST=Department of Medical Sciences Thailand, MICROFLASKU= Microbiology Faculty of Liberal Arts and Science, Kasetsart University

### Preparation of bacterial culture and DNA extraction

The DNA of *L. monocytogenes* was prepared by centrifuging 1 mL of TSB enrichment solution at 9,520 x g for 1 min, followed by three washes with 1 mL sterile deionized water. Then, 100 mL of sterile water was added to pellets and mixed thoroughly. The mixture was incubated in a heat box at 100°C for 10 min, centrifuged at 9,520 x g for 1 min. A pipette was used to transfer the supernatant to purified genomic DNA of *L. monocytogenes* using the DNeasy kit (QIAGEN, Germany) and collected a template of DNA and stored at –20°C for one week.

### LAMP primers design

**Figure-1:**
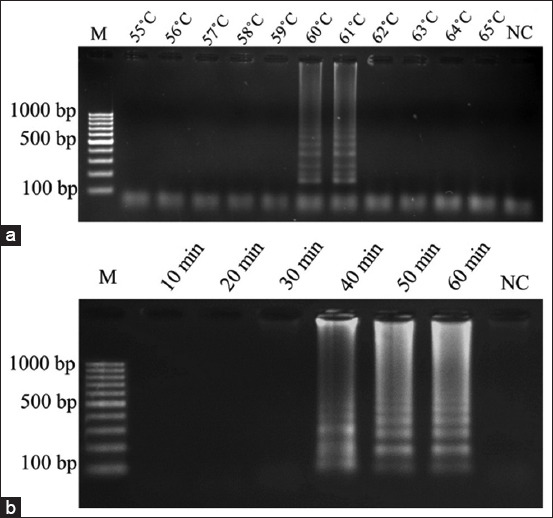
The optimized temperature of the *LMfeoA4* loop-mediated isothermal amplification (LAMP) assay, (a) optimized temperature, (b) optimized time agarose gel electrophoresis profile of LAMP reaction effects of temperature on LAMP reaction. M is DNA 100 bp ladder marker.

The specific iron transport protein gene was used to design primer and classify *L. monocytogenes* by division using the PCR method [7]. The specific region of *feoB* was designed as multiplex PCR primers to detect *L. monocytogenes* and sequenced to confirm and show the identification of bacterial genes were identified 100% of DNA sequence which PCR primers were highly specific with target gene [24]. The 216-bp-specific region of *feoB* was used in this study. To ensure assay specificity, five LAMP primer sets were designed for the target gene (*feoB*). All *feoB* sequences were aligned with CLUSTALW (https://www.genome.jp/tools-bin/clustalw), and the conserved regions were used for analysis across *Listeria* spp. This study designed LAMP primers using PrimerExpoler V5 (http://primerexplorer.jp/lampv5e/index.html) and confirmed the specificity of the designed F2 and B2 primers by prediction using *in silico* PCR amplification (http://insilico.ehu.es/PCR), which indicated that *feoB* detected 19 strains of *L. monocytogenes*, including *L. monocytogenes* 07PF0776, *L. monocytogenes* ATCC19117, *L. monocytogenes* Clip81459, *L. monocytogenes* J1-220, *L. monocytogenes* J1816, *L. monocytogenes* L312, *L. monocytogenes* SLCC2378, *L. monocytogenes* SLCC2540, *L. monocytogenes* SLCC2755, *L. monocytogenes* serotype 4b str.LL195, *L. monocytogenes* serotype 7 str.SLCC2482, *L. monocytogenes* str. 4b F2365, *L. monocytogenes* strain J1776, *L. monocytogenes* strain J1817, *L. monocytogenes* strain J1926, *L. monocytogenes* strain J2-064, *L. monocytogenes* strain J2-1091, *L. monocytogenes* strain N1-011A, and *L. monocytogenes* strain R2-502. Further, no specific target *feoB* primer sets of *Listeria innocua, Listeria ivanovii*, *Listeria* *welshimeri*, and other pathogenic bacteria were detected. The specific LAMP primers, including F3 and B3 (outer primers), forward internal primer (FIP) and backward internal primer (BIP) (inner primers), and all primers were synthesized by Ward Medic IDT (Thailand), as shown in Figure-1.

### Optimization of LAMP reaction conditions

First, each LAMP reaction of the *feoB* genes was performed in a total volume of 25 μL containing 0.8 mM each LAMP inner primers (BIP and FIP), 0.4 mM each LAMP outer primers (B3 and F3), 1.4 mM dNTPs (Thermo Fisher Scientific Inc. USA), 0.8 M Betaine, 4 mM MgSO_4_, 1× of *Bst* polymerase buffer (Biolab Inc. UK), 8 U of *Bst* DNA polymerase, 2 μL of DNA template and added up sterile deionized water to 25 μL. The LAMP reactions were modified to the methods described in the previous report [25]. The optimum temperature was determined using the LAMP condition by various temperatures at 55°C, 56°C, 57°C, 58°C, 59°C, 60°C, 61°C, 62°C, 63°C, 64°C, and 65°C for 60 min and stop the reaction by heating at 90°C for 5 min. The LAMP products were analyzed by 2% AGE. Under the identified optimal temperature, the optimization time was evaluated for 10, 20, 30, 40, 50, and 60 min, and stopped the reaction. Each LAMP product was assessed using 2% AGE. Two microliters of sterile water were used as the negative control (NC).

### Specificity tests of the LAMP assays in pure culture

The specificity of the LAMP assays was determined under the optimized conditions based on the *feoB* gene were tested using the 22 bacterial isolates (Table-1), including *L. monocytogenes* and non-*L. monocytogenes*. Each microbial DNA template was amplified with LAMP using *LMfeoB* primers, and products from amplification were analyzed with 2% AGE. The specificity detection results were compared with conventional culture and PCR assay systems.

### PCR primers and condition

The two outer primers (F3 and B3) of each LAMP primer set were used to amplify the *feoB* genes by PCR. The PCR reaction consisted of 2.5 μM of each outer primer, 2.5-mM dNTPs, 4-mM MgCl_2_, 0.3 U of *Taq* DNA polymerase, and 2-μL DNA templates, which added up dH_2_O to 20 μL. The five steps of PCR cycling were as follows: 5 min for the initial denaturation step at 95°C, followed by 35 cycles of amplification step, including denaturation at 95°C for 30 s. After the denaturation step, the temperature was reduced to 62°C for 30 s (annealing). In the last step (extension), the optimal temperature of 72°C was used for 30 s and 5 min at 72°C at the end of the reaction. Two microliters of each PCR product were examined using 2% AGE, while 2 μL of sterile dH_2_O was included as the NC.

### DNA probe design and LAMP primers combined with hybridization

The probe of DNA was designed using regions in the middle of the FIP and BIP primers for detection by LAMP-LFD. The FIP primer was 5′-labeled with DIG and the probe of DNA was 5′ end-labeled with biotin (Petty patent submission numbers 2003002812). The CLUSTALW program (https://www.genome.jp/tools-bin/clustalw) was used to align the *feoB* nucleotide sequences in the *L. monocytogenes* strain CFSAN023463 (GenBank Accession No: CP012021.1) with the DNA probe.

The primer sets used for LAMP amplification coupled with LFD, FIP was modified using DIG labeling at the 5’ end of the oligonucleotide sequence. Ward Medic IDT (Thailand) synthesized and labeled all primers and DNA probes protected by petty patent submission numbers 2003002812.

### LAMP-LFD assay conditions

**Figure-2:**
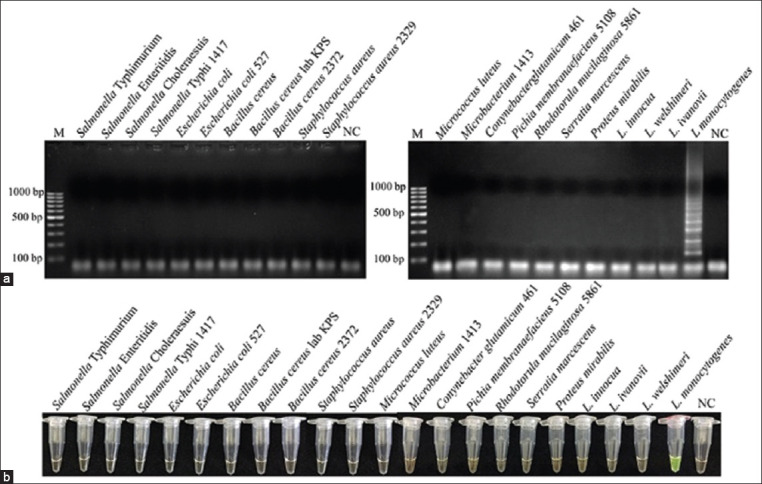
The specificity test of *LMfeoB*4 loop-mediated isothermal amplification (LAMP) primer sets using LAMP-agarose gel electrophoresis (a) and LAMP-*SYBR Green* I (b). Lane M represents 100 bp DNA ladder marker, Lane NC represents negative control (without DNA template).

LAMP combined with hybridization reactions were operated in a 25-μL volume containing 0.8-μM DIG-labeled FIP primer, 0.8-μM BIP primer, 0.4-μM F3 primer, 0.4-μM B3 primer, 1.6-μM of biotin-labeled DNA probe, 0.8 M betaine, 1.4 mM of each dNTPs, 4-mM MgSO_4_, 8 U of the *Bst* DNA polymerase, 1× of the *Bst* buffer, and DNA template 2 mL by boiling method. The mixture of reaction was incubated at 60°C for 60 min. After incubation and stopping the reaction, 8 μL of each hybridized LAMP product was transferred to 100 μL of the assay buffer (Serve Science, Thailand). Finally, a commercial LFD strip (Serve Science) was dipped into the reaction mixture. The result was visualized as a cherry-pink color signal at the control and test lines after 1 min. The control and test lines appeared on the LAMP-LFD showing a positive result. However, the sample producing a single line at the control showed a negative result (Figure-2). If no line appeared at the control line, the test strip could be considered invalid.

### Detection limits of the LAMP-LFD, LAMP, and PCR assays in pure culture

The limit of detection (LOD) for the LAMP-LFD, LAMP-AGE, LAMP-*SYBR Green* I, and PCR assays were evaluated using tenfold serial dilutions of a 12-h culture of *L. monocytogenes* in TSB enrichment solution. To count the colonies number of bacteria in 100 μL aliquots of each tenfold dilution using spread technique in duplicate on palcam agar and colonies number, these plates were counted after incubation for 24 h at 37°C. For DNA extraction, 100 μL of each tenfold dilution 10^8^ colony-forming unit (CFU)/mL to 10^0^ CFU/mL was used to prepare the DNA template using the boiling method, as described above in bacterial culture and DNA extraction methods. Then, 2 μL of the prepared DNA template was added to the LAMP reaction, and the results of LAMP-LFD, LAMP-AGE, and LAMP-*SYBR Green* I were compared with the detection limits of conventional culture and PCR assay systems.

### Detection limits of the LAMP-LFD and LAMP assays in purified DNA

The detection limits of the LAMP-LFD and LAMP-AGE assays were determined using purified DNA from a 12-h pure culture of *L. monocytogenes* in a TSB enrichment solution. After overnight growth, the purified genomic DNA was extracted from L. monocytogenes using a DNeasy kit (QIAGEN). The concentration of the extracted DNA was determined at A260/280 using a spectrophotometer (Nanodrop800, Thermo Scientific) and diluted using 10-fold serial dilutions before adding two microliters of the prepared DNA to the LAMP-LFD and LAMP-AGE reactions.

### Detection limits of the LAMP-LFD, LAMP, and PCR assays for artificial contamination in frozen products with and without pre-enrichment

The pure culture of *L. monocytogenes* was grown in tryptic soy broth, as previously described in detection limits of the LAMP-LFD, LAMP, and PCR assays in pure culture method, and 1 mL of the *L. monocytogenes* suspension was diluted with peptone salt solution to yield cell concentrations ranging from 10^1^ CFU/mL to 10^8^ CFU/mL. Then, 1 mL of diluted *L. monocytogenes* suspension was added to 225 μL half Fraser broth in a stomacher bag for artificial contamination in 25 g chicken meat samples. The chicken meat samples were prepared without adding *L. monocytogenes* as the NC. The inoculated chicken meat samples were homogenized in a stomacher for 90 s. The prepared samples were used without pre-enrichment samples determined by LAMP-LFD, LAMP, PCR assays, and conventional methods. The suspension of prepared samples was incubated at 37°C for 12 h and used with pre-enrichment samples determined by LAMP-LFD, LAMP, PCR assays, and conventional methods.

### Detection of *L. monocytogenes* in frozen product samples

Twenty frozen product samples, including pork, chicken, beef, and fish, were collected from a supermarket in Kanchanaburi Province, Thailand. Five samples of each type of frozen meat were collected. Then, 25 g of each frozen food sample were added to 225 μL half Fraser broth in a stomacher bag. The inoculated samples were homogenized in a stomacher for 90 s and the suspension was incubated at 37°C for 12 h. The boiling method was used to extract DNA from 1 mL of supernatant samples, and each 2 μL of extracted DNA sample was used as a template of DNA for the LAMP-LFD, the LAMP, and PCR assays. All samples were analyzed using the ISO 11290-1 (2017) standard method [26-28].

## Results

### Optimization of LAMP reaction conditions

Five LAMP primer sets were evaluated for *L. monocytogenes feoB* gene fragment detection. The optimum LAMP temperature for amplification of the *LMfeoB*1, *LMfeoB*2, *LMfeoB*3, *LMfeoB*4, and *LMfeoB*5 primer sets was determined. The results with equal LAMP reaction master mix concentrations were used and assessed based on 2% AGE. The optimum temperature for the *LMfeoB*4 primer set was 60°C and 61°C (Figure-1a), whereas no amplification occurred at any optimum temperature for the *LMfeoB*1, *LMfeoB*2, *LMfeoB*3, and *LMfeoB*5 primer sets. Therefore, the result indicates that the LAMP assay using the *LMfeoB*4 primer set was effective for *L. monocytogenes* detection based on *feoB* gene within 60°C and 61°C. Based on the result, LAMP amplicons showed the clearest pattern at 60°C, and thus 60°C was considered the optimal temperature for LAMP assay.

The optimum reaction time for LAMP amplification of the *LMfeoB*4 primer sets was 60 min. Figure-1b depicts the results when equal LAMP reactions at 60°C were used and assessed based on 2% AGE. NC followed no amplification. The results indicated that the LAMP assay using *LMfeoB*4 primer set for the detection of *L. monocytogenes* prosperously amplified the *feoB* genes, and the LAMP products optimized temperatures and time was at 60°C for 60 min.

### Specificity of the LAMP method

Figure-2a depicts the specificity tests of the LAMP-AGE, and LAMP-*SYBR Green* I assay are shown in Figure-2b for *L. monocytogenes* detection using *LMfeoB*4 primer sets was that target *feoB* genes. The results of the *LMfeoB*4 primer set do not cross-amplify target genes in non-*L. monocytogenes* strains. These results indicate that the LAMP-*SYBR Green* I and LAMP-AGE assays based on *LMfeoB*4 primer sets of *feoB* genes are considerably efficient and highly specific for the detection of *L. monocytogenes*.

### Detection limits in pure culture

**Figure-3:**
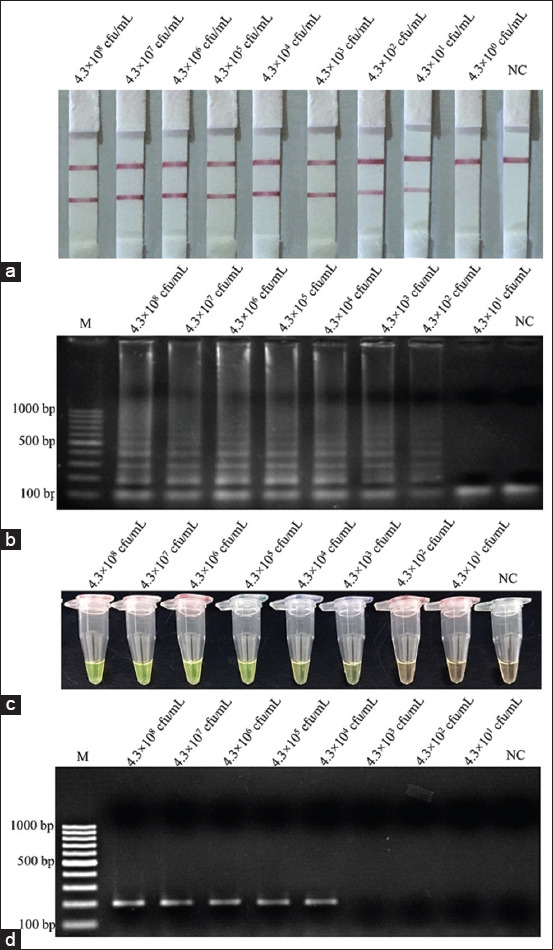
Limit of detection for detection of *Listeria monocytogenes* pure culture using (a) loop-mediated isothermal amplification (LAMP)-lateral flow dipstick, (b) LAMP-agarose gel electrophoresis, (c) LAMP-*SYBR Green* I, (d) polymerase chain reaction. Lane M represents 100 bp DNA ladder marker, Lane NC represents negative control (without DNA template).

The initial concentration for cultures of *L. monocytogenes* containing 4.3×10^8^ CFU/mL were diluted to be 10^7^ CFU/mL, 10^6^ CFU/mL, 10^5^ CFU/mL, 10^4^ CFU/mL, 10^3^ CFU/mL, 10^2^ CFU/mL, and 10^1^ CFU/mL before boiling DNA extraction method and amplification. The results of detection limits of the LAMP-LFD, LAMP-AGE, LAMP-*SYBR Green* I, and PCR assays in pure culture are shown in Figure-3. Although the detection limit of the LAMP-LFD was 43 CFU/mL (Figure-3a) higher than those of LAMP-AGE (Figure-3b), LAMP-*SYBR Green* I (Figure-3c), and PCR assay (Figure-3d), which were 4.3×10^2^ CFU/mL, 4.3×10^3^ CFU/mL, and 4.3×10^4^ CFU/mL, respectively.

### Detection limits in purified DNA

**Figure-4:**
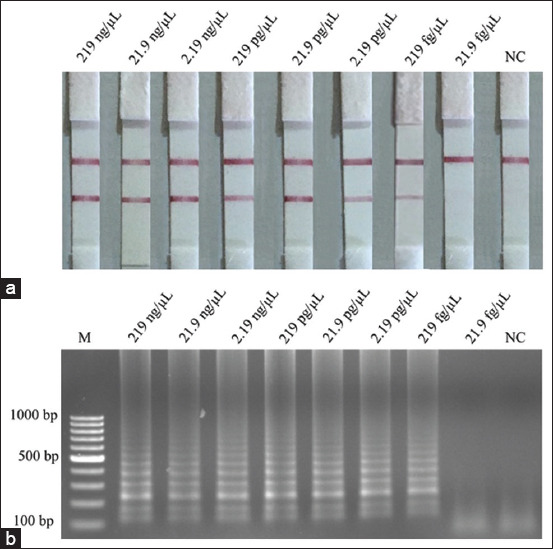
Limit of detection for the detection of purified DNA of *Listeria monocytogenes* using (a) loop-mediated isothermal amplification (LAMP)-agarose gel electrophoresis and (b) LAMP-lateral flow dipstick. Lane M represents 100 bp DNA ladder marker, Lane NC represents negative control (without DNA template).

The concentration of *L. monocytogenes* pure culture at 4.3×10^8^ CFU/mL was extracted and purified. The LOD for the LAMP-LFD (Figure-4a) and the LAMP-AGE assays (Figure-4b) using pure DNA based on the *feoB* gene was 219 fg for both assays (Figure-4). Using ten-fold serial dilutions for pure DNA and grown cells, this study discovered that serial dilution is the most crucial element in determining the detection thresholds for regulatory guidance and the number of standard procedures. The detection limit obtained by utilizing genomic DNA as a DNA template shows that the results are more sensitive than pure grown cells.

### Detection limits in artificial contamination frozen products with and without pre-enrichment

**Figure-5:**
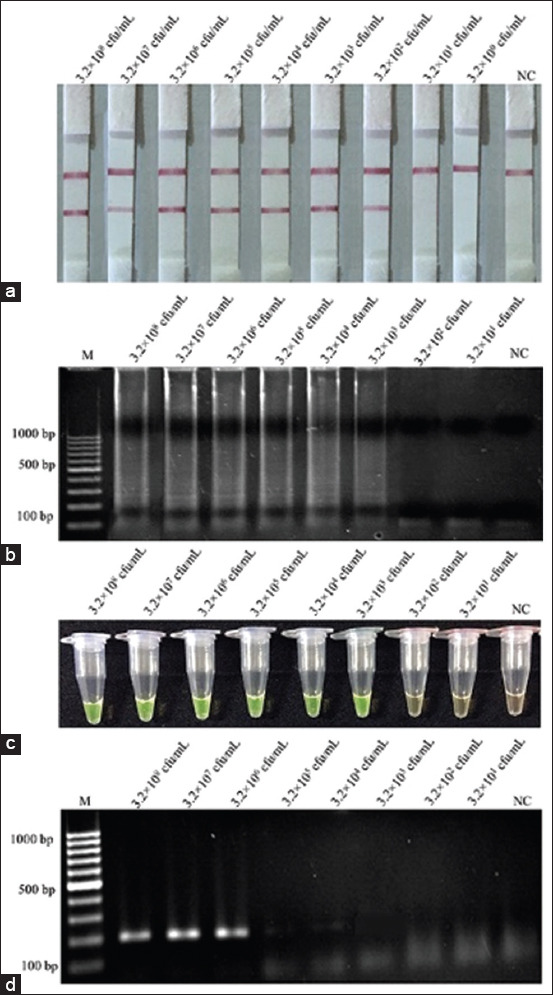
Limit of detection for the detection of *Listeria monocytogenes* in artificially inoculated of chicken frozen food samples with pre-enrichment by using (a) Loop-mediated isothermal amplification (LAMP)-lateral flow dipstick, (b) LAMP-agarose gel electrophoresis, (c) LAMP-*SYBR Green* I and (d) polymerase chain reaction assays. Lane M represents 100 bp DNA ladder marker, Lane NC represents negative control (without DNA template).

**Figure-6:**
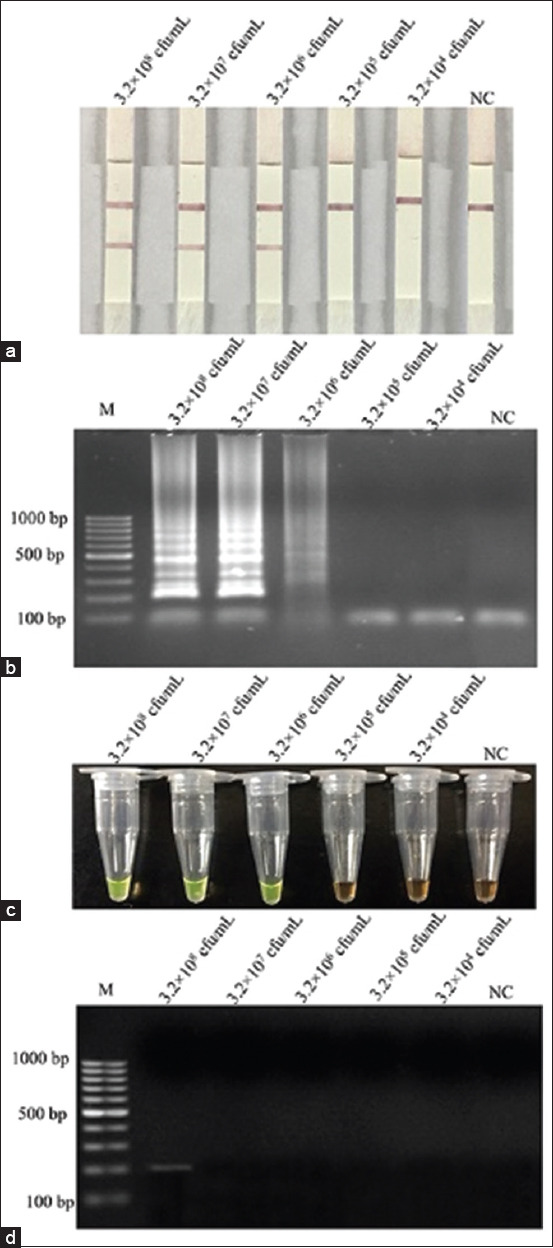
Limit of detection for the detection of *Listeria monocytogenes* in artificially inoculated of chicken frozen food samples without pre-enrichment using (a) loop-mediated isothermal amplification (LAMP)-lateral flow dipstick, (b) LAMP-agarose gel electrophoresis, (c) LAMP-*SYBR Green* I and (d) polymerase chain reaction assays. Lane M represents 100 bp DNA ladder marker, Lane NC represents negative control (without DNA template).

The detection limit for *L. monocytogenes* in artificially contaminated frozen chicken product samples was determined using the LAMP-LFD, the LAMP-AGE, the LAMP-*SYBR Green* I, and PCR assays based on the *feoB* gene. The LOD of the LAMP-LFD (Figure-5a), LAMP-AGE (Figure-5b), LAMP-*SYBR Green* I (Figure-5c), and PCR assays (Figure-5d) with pre-enrichment was 3.2×10^2^ CFU/mL, 3.2×10^3^ CFU/mL, 3.2×10^3^ CFU/mL, and 3.2×10^6^ CFU/mL, respectively (Figure-5). The LOD of LAMP assay, including LAMP-LFD (Figure-6a), the LAMP-AGE (Figure-6b), and the LAMP-*SYBR Green* I (Figure-6c), and PCR assays (Figure-6d) without pre-enrichment were 3.2×10^6^ CFU/mL and 3.2×10^8^ CFU/mL, respectively (Figure-6).

This result suggests that the developed LFD can provide better sensitivity than the LAMP assay and PCR method. The results showed that the LAMP test was more sensitive than the PCR assay, with and without pre-enrichment of approximately 1000 and 100 times, respectively. The LOD for *L. monocytogenes* in the pure state was greater than the LOD of *L. monocytogenes* in artificially inoculated frozen chicken samples. In addition, the pre-enrichment sample increased the detection limit of the LAMP test, indicating that the rich component in samples that might impact sensitivity was reduced.

### Detection of *L. monocytogenes* in frozen product samples

**Figure-7:**
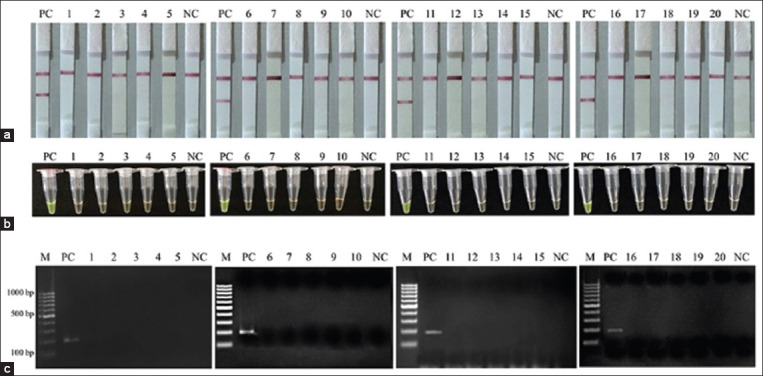
(a-c) The detection results of loop-mediated isothermal amplification (LAMP)-lateral flow dipstick assay based on *feoA* gene of *Listeria monocytogenes* in pork frozen products (1-5), chicken frozen products (6-10), beef frozen products (11-15) and fish frozen products (16-20) against LAMP-*SYBR Green* I assays and polymerase chain reaction. Lane M represents 100 bp DNA ladder marker, Lane PC represents positive control Lane NC represents negative control (without DNA template).

The detection results of the LAMP-LFD assay are based on the *feoB* gene of *L. monocytogenes* in five frozen pork products (1-5), five frozen chicken products (6-10), frozen beef products (11-15), and frozen fish products (16-20). According to culture-based examinations with pre-enrichment, 20 frozen products samples were not contaminated with *L. monocytogenes* (Figure-7). However, the positive control (PC) as artificially inoculated *L. monocytogenes* containing 320 CFU/mL was positive, similar to those identified by the LAMP-LFD, LAMP-*SYBR Green* I, and PCR assays.

## Discussion

In this study, the selection of *feoB* gene was used to detect *L. monocytogenes* because of the key role of home transport and maintenance of intracellular iron homeostasis in the pathogenesis of *L. monocytogenes* [29]. A previous study by Monica and Douglas [7] identified a serotype of *L. monocytogenes* by PCR assay. Three primers were designed from the *L. monocytogenes* genome variable regions and used in combination with the previously described division III primer set to classify 122 strains of *L. monocytogenes* into five serotype groups and *L. monocytogenes* division I, which can interact with only one division of the iron transport protein gene primer. *L. monocytogenes* division I has also been discovered to be the division causing the most severe food poisoning. A study by Awassada [24] compared multiplex PCR to the PCR and conventional culture for detection of pathogenic bacteria in goat milk and bovine milk and detection of *L. monocytogenes*, *Staphylococcus aureus* ATCC25923, *Bacillus cereus*, and *Escherichia coli* ATCC35218. The primer for the detection of *L. monocytogenes* was designed from the *feoB* gene sequence. Recent studies concerning virulence-associated genes, such as *Imo0460*, *prs, PrfA, hly, hlyA, iap, flaA, actA, inlA*, *plcA, plcB, IgY*, and *inlB*, and those involving the pathogenesis of the Listeriosis process have been published [30-32]. The previous studies reported the rapid detection of *L. monocytogenes* foodborne pathogen based on *plc*B and listeriolysin O (*hly*) gene using the LAMP-LFD [25] and LAMP-turbidity assays [33], as shown in Table-2 [7,8,16,17,19,20,21-24,33-35]. The LAMP primers set and DNA probes for *L. monocytogenes* detection were designed based on the *iap* gene using the duplex LFD technique for detecting *L. monocytogenes* in meat products based on LAMP assay [36]. Herein, five primer sets (*LMfeoB*1-*LMfeoB*5) were designed from a specific region of the *feoB* gene on the nucleotide sequence of *L. monocytogenes* strain CFSAN023463 (GenBank accession no ID: CP012021.1). Optimization of LAMP reaction conditions was modified to the previous report by Sirirat *et al*. [25]. Optimization of the LAMP assay was performed on the *FeoB* gene based on the use of four kinds of primers (two inners and two outers) that recognize six distinct regions of a target gene in the presence of *Bst* DNA polymerase, which is an enzyme that can divide double-stranded DNA into single strands without utilizing high temperatures. It can enhance the quantity of target DNA at a single temperature. It is a stable enzyme that functions well at temperatures ranging from 60°C to 65°C. Furthermore, 10× polymerase buffer is a buffer of *Bst* DNA polymerase enzyme used to optimize reaction conditions. It is resistant to diverse contaminants, dNTPs function as subunits in the synthesis of new DNA strands and as substrates in polymerization processes to join the DNA strands until a new strand of DNA is obtained by adding dNTPs to the 3’-OH side. Betaine can speed up the production of DNA polymerase (polymerase activity). It also aids in the disintegration of the double helix structure, stabilizing the linear DNA to make DNA amplification more convenient. This raises the likelihood of raising particular DNA volumes while decreasing base stacking difficulties. Reaction parameters were also optimized by single factor optimization experiment to determine the optimal reaction system of 25 μL as follows: 0.8-μM each LAMP inner primers, 0.4-μM each LAMP outer primers, 1.4-mM dNTPs (Thermo Fisher Scientific Inc.), 0.8 M Betaine, 4-mM MgSO_4_, 1× of *Bst* polymerase buffer (Biolab Inc.), 8 U of *Bst* DNA polymerase, 2 mL of DNA template and added up sterile deionized water to 25 μL. The current study showed the *LMfeoB*4 LAMP reaction food. The most popular gene amplification technique is PCR [7,8], which has amplified the *feoB* gene of *L. monocytogenes* at an optimized temperature of 60°C for 50 min, which was an optimizing time similar to previous study [25]. The optimized time of 50 min for the LAMP reaction was similar to Sirirat *et al*. [25]. These studies observed that the *plc*B and *hly* genes of *L. monocytogenes* used LAMP amplification. For instance, Sudarat *et al*. [36] designed inner and outer primers of LAMP and identified the *iap* gene from *L. monocytogenes* reactions cultured at 65°C for 45 min.

**Table-2:** Comparison of target gene time and detection limits of LAMP-LFD for the detection of the *Listeria monocytogenes* against those of various detection methods.

Method of detection	Target gene	Total time	Detection limit	Reference
PCR	*hlyA*	3 h*	6-60 CFU/mL	[8]
	*PrfA*	24 h	7.5 CFU/25 g	[8]
	*ssrA*	30 h	1-5 CFU/25 g	[8]
	*hlyA*	40 min*	20 CFU/reaction	[35]
	*feoB*	90 min*		[7]
	*feoB*	90 min*		[24]
	*feoB*	90 min*		This study
Real time PCR	*hlyA*	20-48 h	10 CFU/mL	[22]
LAMP	*lmo0460*	50 min*	1.7 CFU/reaction	[20]
	*flaA*	N/A	10 pg and 104 CFUs/reaction	[16]
	*iap*	40 min*	186 CFU/mL	[17]
	*PrfA*	12 h	22 CFU/mL	[19]
	*feoB*	90 min*	219 fg/mL or 4.3 × 10^2^ CFU/mL	This study
LAMP-LFD	*plcB*	90 min*	800 pg/uL and 2.82 × 10^3^ CFU/mL	[35]
	*hlyA*	40 min*	10 pg/mL	[23]
	*iap*	60 min*	800 fg/mL and 20 CFU/mL	[34]
	*feoB*	60 min*	219 fg/mL or 43 CFU/mL	This study
Gold nanoparticle colorimetric biosensing	*plcB*	60 min*	800 pg/uL and 2.82 CFU/mL	[33]
Aptamer-Magnetic colorimetric	*IgY*	N/A	10 CFU/mL	[20]
Aptamer - colorimetric biosensing	*hlyA*	N/A	48.4 ng	[21]

* Exclude the cultured enrichment time. N/A = Data not available, h = Hour, g = Gram, fg = Femtogram genomic DNA, pg = Picogram genomic DNA, ng = Nanogram genomic DNA, min = Minute, CFU/mL = Colony-forming unit per milliliter, LAMP-LFD = Loop-mediated isothermal amplification-lateral flow dipstick, PCR = Polymerase chain reaction

The specificity result of the LAMP assay based on LAMP-AGE and LAMP-*SYBR Green* I in this study showed high specificity by non-amplified product with three other *Listeria* species and 18 non-*Listeria* bacterial strains. However, other *Listeria* species include *L. ivanovii*, *L. innocua, L. welshimeri*, and non-*Listeria* strains include *Salmonella* Typhimurium*, Salmonella* Enteritidis, *Salmonella* Choleraesuis, *Salmonella* Typhi 1417, *E. coli, E. coli* 527*, B. cereus*, *B. cereus* lab KPS*, B. cereus* 2372*, S. aureus, S. aureus* 2329*, Micrococcus luteus, Microbacterium* 1413, *Conynebacter glutamicum* 461*, Pichia membranaefaciens* 5108*, Rhodotorula mucilaginosa* 5861*, Serratia marcescens*, and *Proteus mirabilis*. Following the same procedure as the previous study [25], genomic DNA from each of the 35 bacterial isolates was purified and used for LAMP-LFD by amplifying the *plc*B gene, revealing that the method was could identify *L. monocytogenes* without false positives or cross-reaction with another *Listeria spp*. and non- *Listeria* spp, such as *Campylobacter* spp., *Salmonella* ssp., *E. coli* ATCC 25922, *Shigella* spp., *B. cereus*, *Pseudomonas aeruginosa* ATCC27853, *S. aureus* ATCC25923, *M. luteus*, *Serratia marcescens*, *Citrobacter diversus*, *Klebsiella oxytoca, and Enterobacter aerogenes*.

The result of the LOD of the LAMP and LAMP-LFD assay using purified genomic DNA was 219 fg/mL for both the LAMP and LAMP-LFD assays. The LOD of the LAMP and LAMP-LFD assay in pure culture was 4.3×10^2^ CFU/mL and 43 CFU/mL, respectively. Further, the LOD of LAMP-LFD assay using artificially inoculated chicken in frozen food samples with pre-enrichment was 3.2×10^2^ CFU/mL, and without pre-enrichment was 3.2×10^6^ CFU/mL. These studies revealed that the LOD of *L. monocytogenes* in the pure state was greater than the LOD of *L. monocytogenes* in artificial inoculated frozen chicken samples, which could be due to the fat inhibitors found in food samples. The increase in LAMP-LFD sensitivity could be because LAMP-negative samples generated positive results with the LAMP-LFD method. The sensitivity of LAMP-LFD one order of magnitude higher than that of LAMP when testing pure culture medium might be because the DNA probe is designed based on the nucleotide sequence of the target gene in a reaction, LFD is more specific than the LAMP approach. In the previous studies, Bauer *et al*. [37] and Demmel *et al*. [38] used the labeling agent in the PCR method to improve its sensitivity. According to other studies, the detection limit of LAMP-LFD was close to our study, with a reported detection limit of 2.82×10^0^ CFU/mL for the detection of *L. monocytogenes* based on the *plcB* gene [25,33] and the detection limit of DNA extraction was 4.3×10^2^ CFU/mL. Sudarat *et al*. [36] reported that the detection limit of the PCR assay was ten-fold lower than the LAMP assays. The detection limit of the LAMP-AGE and LAMP-LFD assays using purified genomic DNA and pure culture was 800 fg based on the *iap* gene and 900 fg based on *prs* gene, with a pure culture of 20 CFU/mL [34]. These results indicated that the detection limit using purified genomic DNA has higher sensitivity than pure cultured. The enrichment step is very helpful for detecting *L. monocytogenes* in frozen food products because it multiplies viable cells, reduces interference from powdered substances through dilution, and eliminates the issue of false-positive results. Dead cells can cause false-positive results, which become negligible after enrichment [39].

The result of evaluating artificial contamination with and without pre-enrichment of *L. monocytogenes* in frozen chicken products indicates that LAMP-LFD, LAMP-*SYBR Green* I, and PCR demonstrate 100% accuracy compared to conventional methods and PCR. The LAMP primer *LMfeoB*4 detected *L. monocytogenes* in 20 frozen food products and showed positive results in all four PC samples, yielding the same detection as the conventional method, while the PCR detected *L. monocytogenes* contamination in 20 samples with negative results. The LAMP-LFD was shown to be more sensitive than LAMP-AGE, LAMP-*SYBR Green* I, and PCR assay (Table-3). Eventually, LAMP-LFD revealed no false positives in any of the 20 samples of frozen food products. The LAMP-LFD demonstrated higher accuracy than conventional culture methods, LAMP-*SYBR Green* I, and PCR assay. The LAMP-LFD test was created to detect DIG-labeled LAMP products hybridized to a biotin-labeled specific DNA probe. Then, a gold-labeled anti-biotin antibody detected the biotin-labeled specific DNA probe. This triple-labeled complex was ultimately avidin-trapped at a test line, resulting in a cherry-pink band (positive result). Non-LAMP products, on the other hand, hybridized with the biotin-labeled specific probe and bound the gold-labeled anti-biotin antibody but did not bind avidin due to a lack of biotin; thus, this complex passed through to the test line but was detained at the control line. By comparison, LAMP-LFD has two advantages over LAMP-AGE. First, it saves time and prevents the use of carcinogens, such as ethidium bromide in AGE. The LAMP products can easily be detected by dipping the strips into assay buffer, reducing the total time to <40 min. In addition, the specificity and sensitivity of the LAMP assay increased by hybridization with a specific probe for LAMP amplicons.

**Table-3:** Comparison of sensitivity, specificity, accuracy, coefficient of variation, and process time of LAMP-LFD for detection of *L. monocytogenes* in frozen food products (pork, chicken, beef, and fish meat) against standard culture, PCR, and LAMP-*SYBR Green* I assays.

Diagnosis methods	Positive results of *L. monocytogenes*	Sensitivity (%)	Specificity (%)	Accuracy (%)	Coefficient of variation (%)	Process time
LAMP-LFD	4	100	100	100	0	1.00 h
LAMP-*SYBR Green* I	4	100	100	100	0	1.00 h
PCR	4	100	100	100	0	2.30 h
Culture	4	100	100	100	0	5-7 d

LAMP-LFD=Loop-mediated isothermal amplification-lateral flow dipstick, PCR=Polymerase chain reaction, *L. monocytogenes=Listeria monocytogenes*

Furthermore, the combination of LAMP and LFD was faster than the traditional methods of PCR, which requires 2-3 h for thermal cycling [40]. The LAMP- LFD was proven to be significantly specific, sensitive, convenient, fast, and accurate. The conventional method for detecting *L. monocytogenes* is a labor-intensive procedure that takes about 4-5 days and requires numerous subculture phases, complex biochemical and serological tests. It is time-consuming but has excellent accuracy for *L. monocytogenes* live cells according to the ISO 11290-1 (2017) standard method [26] for foods that support the growth of *L. monocytogenes*, the previous limit of not detected in 25 g for foods PCR and real-time monitoring. In most cases, PCR takes 1-2 h and requires the use of a thermocycler to generate target DNA; about 20 times less DNA is produced [41].

## Conclusion

This study developed LAMP-LFD as a rapid screening test for detecting *L. monocytogenes* using a specific region of the *feoB* gene; the LAMP-LFD was highly sensitive, specific, and accurate. Consequently, the technique would be a valuable tool for the rapid screening and monitoring of *L. monocytogenes* contamination in frozen food products. There are limitations to this test kit, 12 h enrichment is required. If the DNA content is 219 fg/µL then a positive result will be shown and low sensitivity can easily result in contamination and consequently in false-positive.

## Authors’ Contributions

WN and WS: Carried out the study, performed the analysis of data, and drafted the manuscript. WN and CS: Study conception, study design, and reviewed the manuscript. WN and WS: Revised the manuscript. All authors have read and approved the final manuscript.

## References

[1] FDA. Bad Bug Book-Foodborne Pathogenic Microorganisms and Natural Toxins (2012). Department of Health and Human Services, US.

[2] Authority E.F.S (2021). The European union one health 2019 zoonoses report. Efsa J.

[3] Pohl A.M, Pouillot R, Bazaco M.C, Wolpert B.J, Healy J.M, Bruce B.B, Laughlin M.E, Hunter J.C, Dunn J.R, Hurd S, Rowlands J.V, Saupe A, Vugia D.J, van Doren J.M (2019). Differences among incidence rates of invasive listeriosis in the U.S. foodnet population by age, sex, race/ethnicity, and pregnancy status, 2008-2016. Foodborne Pathog. Dis.

[4] Tack D.M, Ray L, Griffin P.M, Cieslak P.R, Dunn J, Rissman T, Jervis R, Lathrop S, Muse A, Duwell M, Smith K, Tobin-D'Angelo M, Vugia D.J, ZablotskyKufel J, Wolpert B.J, Tauxe R, Payne D.C (2020). Preliminary incidence and trends of infections with pathogens transmitted commonly through food-foodborne diseases active surveillance network, 10 U.S. sites, 2016-2019. MMWR Morb. Mortal. Wkly. Rep.

[5] CDC. Surveillance for Foodborne Disease Outbreaks, United States, 2015 (2017). CDC Atlanta, Georgia.

[6] FAO/WHO (2004) Risk Assessment of *Listeria monocytogenes* in Ready-to-Eat Food. Interpretive Summary WHO, Geneva.

[7] Monica K.B, Douglas R.C (2003). *Listeria monocytogenes* serotype identification by PCR. J. Clin. Microbiol.

[8] Chen J.Q, Healey S, Regan P, Laksanalamai P, Hu Z (2017). PCR-based methodologies for detection and characterization of *Listeria monocytogenes* and *Listeria ivanovii* in foods and environmental sources. Food Sci. Human Well.

[9] Piet A, Rijn V, Boonstra J (2021). Critical parameters of real-time reverse transcription-polymerase chain reaction (RT-PCR) diagnostics:Sensitivity and specificity for bluetongue virus. J. Virol. Methods.

[10] Green M.R, Sambrook J (2019). Nested Polymerase Chain Reaction (PCR). Cold Spring Harb. Protoc.

[11] Zhenzhen W, Miho S, Yen T.H.N, Yayoi H, Nariaki N, Haruhiko M, Ayako Y (2018). Development of nested multiplex polymerase chain reaction (PCR) assay for the detection of *Toxocara canis*, *Toxocara cati,* and *Ascaris suum* contamination in meat and organ meats. Parasitol. Int.

[12] Kawasaki S, Pina F.M, Naoko K.H, Yukio O, Kazuko T, Takashi S, Shinichi K (2011). Development of the multiplex PCR detection kit for *Salmonella* spp., *Listeria monocytogenes*, and *Escherichia coli* 0157:H7. Japan Agric. Res. Q.

[13] Rio L.E.J, Miguel A.P (2021). SYBR-green real-time PCR assay with melting curve analysis for the rapid identification of *Mytilus* species in food samples. Food control.

[14] Notomi T, Okayama H, Masubuchi H, Yonekawa T, Watanabe K, Amino N, Hase T (2000). Loop-mediated isothermal amplification of DNA. Nucleic Acids Commun.

[15] Hong T.C, Mai Q.L, Cuong D.V, Parida M, Minekawa H, Notomi T, Hasebe F, Morita K (2004). Development and evaluation of a novel loop-mediated isothermal amplification method for rapid detection of severe acute respiratory syndrome coronavirus. J. Clin. Microbiol.

[16] da Costa A.P.R, de Lira Nunes M, Mendes-Marques C.L, de Almeida A.M.P, Leal N.C (2014). Loop-mediated isothermal amplification (LAMP) for the detection of *Listeria monocytogenes* and major pathogenic serotype. Am. J. Anal. Chem.

[17] Wang D, Huo G, Ren D, Li Y (2010). Development and evaluation of a loop-mediated isothermal amplification (LAMP) method for detecting *Listeria monocytogenes* in raw milk. J. Food Saf.

[18] Mori Y, Nagamine K, Tomita N, Notomi T (2000). Detection of loop-mediated isothermal amplification reaction by turbidity derived from magnesium pyrophosphate formation. Biochem. Biophys. Res. Commun.

[19] Cho A.R, Dong H.J, Seo K.H, Cho S (2014). Development of a loop-mediated isothermal amplification assay for detecting *Listeria monocytogenes prfA* in milk. Food Sci. Biotechnol.

[20] Yushen L, Wang J, Song X, Xu K, Chen H, Zhao C, Li J (2018). Colorimetric immunoassay for *Listeria monocytogenes* by using core gold nanoparticles, silver nanoclusters as oxidase mimetics, and aptamer-conjugated magnetic nanoparticles. Microchim. Acta.

[21] Du J, Singh H, Dong W, Bai Y, Yi T.H (2018). Colorimetric detection of *Listeria monocytogenes* using one-pot biosynthesized flower-shaped gold nanoparticles. Sensors Actuat. B Chem.

[22] Lalle M, Possenti A, Dubey J.P, Pozio E (2018). Loop-mediated isothermal amplification-lateral-flow dipstick (LAMP-LFD) to detect *Toxoplasma gondii* oocyst in the ready-to-eat salad. Food Microbiol.

[23] Cheng N, Xu Y, Yan X, Shang Y, Zhu P, Tian W, Liang Z, Xu W (2016). An advance visual qualitative and Eva green-based quantitative isothermal amplification method to detect *Listeria monocytogenes*. J. Food Saf.

[24] Awassada P (2015). Comparison of Conventional Culture and PCR for Detection of Pathogenic Bacteria in Goat Milk and Bovine Milk and Detection of *Listeria monocytogenes*, *Bacillus cereus*, *Staphylococcus aureus* ATCC25923 and *Escherichia coli* ATCC35218 Using Multiplex PCR. M.Sc. Thesis, Faculty of Agriculture at Kamphaeng Saen, Kasetsart University. Nakhon Pathom, Thailand.

[25] Sirirat W, Thayat S, Supatra A, Thongchai K, Pichapak S, Somchai S, Kosum C (2017). Development of a rapid screening test for *Listeria monocytogenes* in raw chicken meat using loop-mediated isothermal amplification (LAMP) and lateral flow dipstick (LFD). Food Anal. Methods.

[26] International Organization for Standardization. Microbiology of Food Feeding Stuffs-Horizontal Method for the Detection and Enumeration of *Listeria monocytogenes* (2017). International Organization for Standardization, Switzerland.

[27] Gnanou B.N, Lombard B, Guillier L, François D, Romero K, Pierru S, Bouhier L, Rollier P (2019). Validation of standard method EN ISO 11290-Part 1-de-tection of *Listeria monocytogenes* in food. Int. J. Food Microbiol.

[28] Rollier P, Lombard B, Guillier L, François D, Romero K, Pierru S, Bouhier L, Besse N (2018). Validation of Standard Method EN ISO 11290-Part 2 for the Enumeration of *Listeria monocytogenes* in Food. Int. J. Food Microbiol.

[29] Lechowicz J, Krawczyk-Balska A (2015). An update on the transport and metabolism of iron in *Listeria monocytogenes*:The role of proteins involved in pathogenicity. Biometals.

[30] Liu Z, Zhu J, Xia X, Wang L, Yang C, Li X (2015). Development of a loop-mediated isothermal amplification assay based on lm0460 sequence for detection of *Listeria monocytogenes*. J. Food Saf.

[31] Zeinali T, Jamshidi A, Rad M, Bassami M (2015). A comparison analysis of *Listeria monocytogenes* isolates recovered from chicken carcasses and humans by using RAPD PCR. Int. J. Clin. Exp. Med.

[32] Day J.B, Basavanna U (2015). Real-time PCR detection of *Listeria monocytogenes* in infant formula and lettuce following macrophage-based isolation and enrichment. J. Appl. Microbiol.

[33] Wachiralurpan S, Sriyapai T, Areekit S, Sriyapai P, Augkarawaritsawong S, Santiwatanakul S, Chansiri K (2018). Rapid colorimetric assay for detection of *Listeria monocytogenes* in Food samples using LAMP formation of DNA concatemers and gold nanoparticles-DNA probe complex. Front. Chem.

[34] Ledlod S, Areekit S, Santiwatanakul S, Chansiri K (2020). Colorimetric aptasensor for detecting *Salmonella* spp., *Listeria monocytogenes*, and *Escherichia coli* in meat samples. Food Sci. Technol. Int.

[35] Wachiralurpan S, Sriyapai T, Areekit S, Sriyapai P, Thongphueak D, Santiwatanakul S, Chansiri K (2017). A one-step rapid screening test of *Listeria monocytogenes* in food samples using a real-time loop-mediated isothermal amplification turbidity assay. Anal. Methods.

[36] Sudarat L, Kespunyavee B, Supatra A, Somchai S, Kosum C (2020). Development of a duplex lateral flow dipstick test for the detection and differentiation of *Listeria* spp. and *Listeria monocytogenes* in meat products based on loop-mediated isothermal amplification. J. Chromatogr. B Analyt. Technol. Biomed. Life Sci.

[37] Bauer T, Kirschbaum K, Panter S, Kenk M, Bergemann J (2011). Sensitive detection of soy (*Glycine max*) by real-time polymerase chain reaction targeting the mitochondrial *atpA* gene. J. AOAC Int.

[38] Demmel A, Hupfer C, Ilg Hampe E, Busch U, Engel K.H (2008). Development of a real-time PCR for the detection of lupine DNA (*Lupinus* species) in foods. J. Agric. Food Chem.

[39] Wang L, Zhao P, Si X, Li J, Dai X, Zhang K, Gao S, Dong J (2020). Rapid and specific detection of *Listeria monocytogenes* with an isothermal amplification and lateral flow strip combined method that eliminates false-positive signals from primer-dimers. Front. Microbiol.

[40] Kumvongpin R, Jearanaikoon P, Wilailuckana C, Sae Ung N, Prasongdee P, Daduang S, Wongsena M, Boonsiri P, Kiatpathomchal W, Swangvaree S.S, Daduang J (2017). Detection assay for HPV16 and HPV18 by loop-mediated isothermal amplification with lateral flow dipstick tests. Mol. Med. Rep.

[41] Mashooq M, Kumar D, Niranjan A.K, Agarwal R.K, Rathore R (2016). Development and evaluation of probe-based real-time loop-mediated isothermal amplification for *Salmonella*:A new tool for DNA quantification. J. Microbiol. Methods.

